# Simulating NIRS and MRS Measurements During Cerebral Hypoxia-Ischaemia in Piglets Using a Computational Model

**DOI:** 10.1007/978-1-4939-0620-8_25

**Published:** 2014-03-22

**Authors:** T. Hapuarachchi, T. Moroz, A. Bainbridge, S. Faulkner, D. Price, K. D. Broad, D. Thomas, E. Cady, X. Golay, Nicola Robertson, Ilias Tachtsidis

**Affiliations:** 1 CoMPLEX, University College London, London, UK; 2 Department of Medical Physics and Bioengineering, University College London, Room 3.18, Malet Place Engineering Building, Gower Street, London, WC1E 6BT UK; 3 Medical Physics and Bioengineering, University College London Hospitals, London, UK; 4 Institute for Women’s Health, University College London, London, UK; 5 Institute of Neurology, University College London, London, UK

**Keywords:** MRS, Neonatal, Mathematical modelling, Intracellular pH, Parameter optimisation

## Abstract

We present a group analysis of the changes in cerebral haemodynamics, and the oxidation state of cytochrome-c-oxidase measured using broadband near-infrared spectroscopy (NIRS) and intracellular pH measured by phosphorous (^31^P) magnetic resonance spectroscopy (MRS) during and after cerebral hypoxia-ischaemia (HI) in 15 piglets. We use a previously published computational model of cerebral metabolism in the piglet [1] to integrate these measurements and simulate HI. We successfully simulate changes in cellular metabolism including shifts in intracellular pH observed in the piglet brain during HI. In this process, we optimise physiological parameters in the model identified through sensitivity analysis (such as the rate of glucose metabolism and intracellular lactate concentration), to fit simulated and measured data. The model fits the data reasonably and suggests a 20 % drop in glucose consumption, a ~65 % increase in lactate concentration and ~35 % drop in the cerebral metabolic rate of oxygen (CMRO_2_) during HI.

## Introduction

Piglets are often used in pre-clinical studies to investigate the effect of physiological intra-partum cerebral HI in mammalian neonates. Using a mathematical and computational model of cerebral metabolism and blood flow, we aim to better understand the complex changes in the brain during these events. The neonatal piglet brain computational model (BrainPiglet) [1] incorporates and simulates NIRS and MRS measurements. These two non-invasive methods are used to monitor brain tissue oxygenation, haemodynamics and metabolism. BrainPiglet was developed from an earlier adult brain model (BrainSignals) [2]—extended to simulate MRS and adapted to the piglet brain. We have recently expanded our model to simulate intracellular pH, considered to be an important biomarker of cerebral pathology [3]. Brain functions are sensitive to changes in pH as the latter affects protein structure. We further optimise physiological parameters in our model to produce the best simulations that fit the measurements. This is vital when using the model with clinical data as biological parameters can vary with alterations in cerebral pathology. These modifications can convey some information about the physiological changes that occur during HI. In this paper we present averaged measurements of cerebral oxygenation, oxidised cytochrome-c-oxidase (oxCCO) level and intracellular pH from 15 piglets and compare these with optimised simulations from our BrainPiglet model.

## Experimental Methods and Protocol

In this study, 15 1-day-old piglets were mechanically ventilated and anaesthetised. Inflatable occluders were surgically inserted around the carotid arteries. Normal levels of arterial oxygen and carbon dioxide, blood glucose and heart rate were maintained. Experiments were under UK Home Office Guidelines (Animals [Scientific Procedures] Act, 1986) and approved by the Institute of Neurology, University College London. Changes (∆) in concentrations of oxy- and deoxy-haemoglobin (∆HbO_2_, ∆HHb) and brain oxCCO (∆oxCCO) were monitored using broadband NIRS. We used ^31^P-MRS to measure changes in concentrations of metabolites such as inorganic phosphate (Pi), phosphocreatine (PCr) and nucleotide triphosphate (NTP; mainly adenosine triphosphate (ATP)). We also estimated intracellular pH using the ^31^P–MRS chemical shifts of Pi and phosphoethanolamine (PEt) [4]. Comparable with normal clinical practice, we continuously recorded systemic variables: arterial blood pressure (P_a_), arterial oxygen saturation (SaO_2_), breathing rate and heart rate. Firstly baseline ^31^P-MRS and NIRS were recorded. Transient HI was then induced (for ~1 h) by inflating the occluders and reducing fractional inspired oxygen (FiO_2_) to 12 % from a normal value of 21 %. Once β-NTP had reduced to ~40 % of baseline, FiO_2_ was gradually returned to normal. This titration was completed over 10–20 min and the carotid occluders were then released. ^31^P-MRS and NIRS were acquired simultaneously every 1 min throughout HI and for a further ~2 h to monitor recovery from HI [4].

## Group Analysis of Measurements

**Fig. 25.1:**
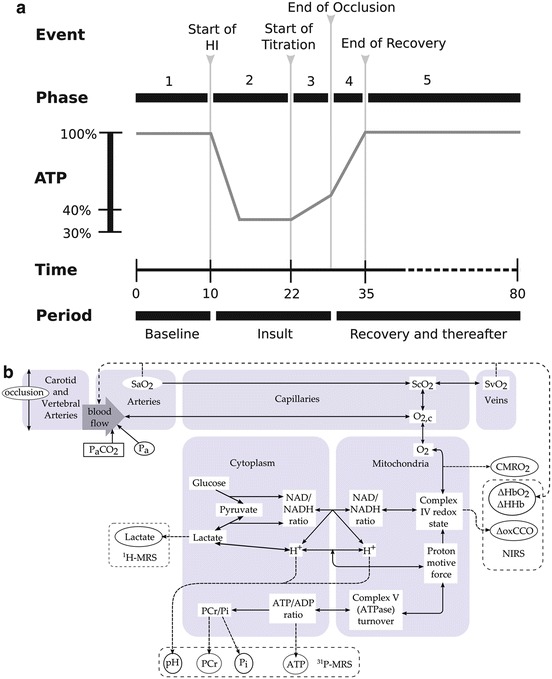
(**a**) Method for averaging data from 15 piglets—the signals were manually divided into five phases. (**b**) Schematic of the BrainPiglet model

Measurements from experiments in 15 piglets that recovered following HI are presented in this paper. We examined ∆HbO_2_, ∆HHb and ∆oxCCO and variations in intracellular pH. We used the difference between ∆HbO_2_ and ∆HHb as a guide to manually divide the data into five phases: (i) baseline, (ii) start of HI to start of FiO_2_ titration, (iii) FiO_2_ titration period, (iv) release of occluders and recovery, and (v) post recovery from HI. Each phase was subsequently divided into ten sections and data in each section were averaged. The mean of each section across all piglets was calculated. This process was repeated for each measurement variable. The time period of each phase was also averaged across all piglets (Fig. 25.1a).

## Model

BrainPiglet is focused on the physiology of the brain [1], and uses differential equations and algebraic relations to simulate cerebral metabolic activity. This model is complex, incorporating ~100 parameters and ~25 variables. It uses P_a_, SaO_2_, arterial carbon dioxide partial pressure (P_a_CO_2_) and the time of carotid occlusion as inputs to simulate NIRS measurements, such as ∆HbO_2_, ∆HHb and ∆oxCCO, and MRS measurements such as Pi, PCr, ATP levels and intracellular pH. It also predicts changes in unmeasured quantities such as CMRO_2_, CBF and intracellular lactate concentration. A simple schematic of the model is in Fig. 25.1b.

## Results

**Fig. 25.2:**
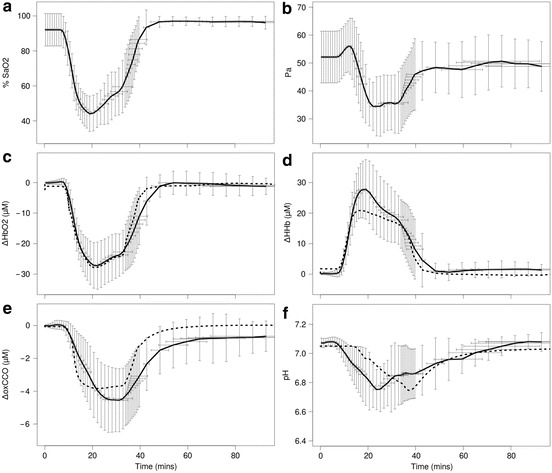
Averaged arterial oxygen saturation (SaO_2_, **a**) and blood pressure (P_a_, **b**) measurements are used as inputs to the model; Averaged NIRS and MRS measurements (*solid line*) compared with modelled results (*dotted line*): oxyhaemoglobin (∆HbO_2_, **c**), deoxyhaemoglobin (∆HHb, **d**), oxidised cytochrome-c-oxidase (∆oxCCO, **e**) and intracellular pH (**f**). HI starts at ~10 min

**Fig. 25.3:**
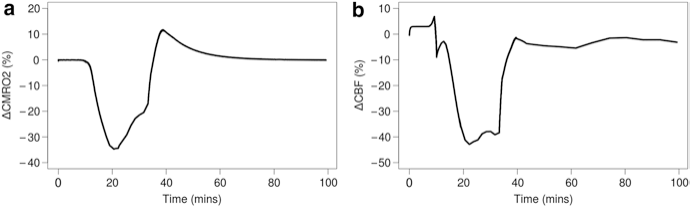
Modelled changes in cerebral metabolic rate of oxygen (∆CMRO_2_, **a**) and cerebral blood flow (∆CBF, **b**). HI starts at ~10 min

Averaged data together with standard deviations are presented in Fig. 25.2. Averaged SaO_2_ and Pa were input into the model. PaCO_2_ was not recorded, however, as the piglets were ventilated, we assumed PaCO_2_ remains constant at 40 mmHg. We used the Morris method [5] to identify the three most influential parameters for ∆HbO_2_, ∆HHb, ∆oxCCO and pH. These parameters were then optimised using the PSwarm method [6] to better fit the model to the data. Optimised parameters are presented in Table 25.1 with percentage change compared to their normal values. Also included is the Morris method ranking of influence for the variables we are investigating (1 = most influential). Optimised model simulations are presented in Fig. 25.2. Simulated CMRO_2_ and cerebral blood flow (CBF) are illustrated in Fig. 25.3.

**Table 25.1:** Optimised parameter values and their influence ranked by the Morris method

Parameter	Optimised value	Percentage change (%)	∆HbO_2_ rank	∆HHb rank	∆oxCCO rank	pH rank
Cytochrome-c-oxidase concentration	0.007 mM	+218			1	
Normal cytoplasm lactate concentration	5.00 mM	+66.7			3	2
Normal blood total haemoglobin	6.35 mM	+17.6	1	1	7	6
Normal arterial blood pressure	53.7 mmHg	+7.40	7	3		
Normal extra-mitochondrial pH	7.05	+0.007			6	1
Autoregulation constant	1.00	No change	3	4		
Special radius in the elastic tension relationship	0.0117 cm	−7.14	2	3		
Normal oxidised fraction of Cu_A_	0.678	+1.19			2	
Normal blood fraction flowing through carotid arteries	0.64	−20.0		2	5	5
Normal glucose metabolism rate	0.00352 mM/s	−20.0			8	3

## Discussion

NIRS and MRS measurements offer a valuable insight into the haemodynamic and metabolic events that occur during cerebral HI. Although some large and significant standard deviations (Fig. 25.2) were observed in the measurements, especially during HI, the general trend in these signals is consistent. During HI we observe the expected drop in SaO_2_ and ∆HbO_2_ and rise in ∆HHb. These concentrations recover very well post HI and return to baseline levels. ∆oxCCO decreases during HI, but does not recover so well and fails to return to the baseline after the insult. Just after the start of HI there is a slight rise in Pa before the major drop during HI. Pa also exhibits significant variation during recovery. While the drop in pH during HI is consistent in all the piglets monitored, there is a lot of variation during both recovery and in the time at which recovery commences. In two piglets we observed an increase in the total haemoglobin concentration during HI. This may be due to back circulation of blood through the vertebral arteries during carotid occlusion. However, these piglets exhibited the same variations in the other metabolic quantities we monitored.

We optimised model parameters to get the best fit of model simulations to in-vivo measurements. We decreased the rate of glucose metabolism by 20 % and increased the normal concentration of cytoplasmic lactate by ~65 %. This suggests an increase in anaerobic respiration as expected during HI. We significantly increased the concentration of oxCCO. However, the normal model oxCCO concentration (0.0022 mM) is quite low and will be updated in future versions of the model. We increased the normal total haemoglobin concentration in blood and decreased the fraction of blood flowing through carotid arteries. This may well occur during HI as the carotid arteries are occluded and the vertebral arteries operate at full capacity. The autoregulation constant in the model represents the capacity of the brain to autoregulate, and ranges from 1.0 in a normal brain to 0.0 in the extreme case. The autoregulation capacity of the piglet remained at 1.00 with optimisation, suggesting that these piglets that recovered following HI are able to autoregulate. Small changes in other parameter values were insignificant. ∆HbO_2_ and ∆HHb are simulated very well during HI. While ∆oxCCO compares well with the measurement, a steeper drop in ∆oxCCO is modelled. The model also simulates a better recovery to baseline than observed in vivo. Modelled intracellular pH reaches an acidity during HI similar to that observed in the piglets, but has a more delayed response. This is caused by a buffering mechanism in the model which we are now investigating. Simulated CMRO_2_ shows ~35 % drop during HI (Fig. 25.3a). This is contradictory to the long-term drop in CMRO_2_ observed in piglets by others [7], suggesting that the current dynamics of our model do not account for the persistent reduction. The model has not taken into account cell death during HI, which may contribute to this effect. Simulated CBF also shows ~40 % drop during HI, and a recovery back to baseline (Fig. 25.3b). We must note that parameter optimisation was conducted on measurements over the complete experiment. However, as the physiological state of the brain before and after HI is quite different, we expect, and are currently attempting, to find distinct sets of parameters that suit each stage of the experiment. This will give us a better insight into the physiological changes that occur between these stages. Further, the distribution of the group data was not taken into account in the simulations.

The BrainPiglet model combines our knowledge of cerebral metabolism and experimental data to explore the effect of HI on brain physiology. It enables informed and practical predictions about patient outcome. The model is able to satisfactorily emulate changes in pH and concentrations of haemoglobin and oxCCO observed during HI. However, while recovery of cerebral haemodynamics and oxygenation is predicted in the model, the metabolic response is not well simulated. This may result from the differing spatial distribution and sensitivity of NIRS and MRS signals—involving cortical and deep white matter, respectively—and the presence of blood in addition to brain tissue in the interrogated tissue volume. Model simulations are also likely to improve with the optimisation techniques outlined above, which will provide more detailed information.
